# Effect of Gaseous Ozone on *Listeria monocytogenes* Planktonic Cells and Biofilm: An In Vitro Study

**DOI:** 10.3390/foods10071484

**Published:** 2021-06-26

**Authors:** Felice Panebianco, Selene Rubiola, Francesco Chiesa, Tiziana Civera, Pierluigi Aldo Di Ciccio

**Affiliations:** Department of Veterinary Sciences, University of Turin, Largo Braccini 2, Grugliasco, 10095 Turin, Italy; felice.panebianco@unito.it (F.P.); selene.rubiola@unito.it (S.R.); tiziana.civera@unito.it (T.C.); pierluigialdo.diciccio@unito.it (P.A.D.C.)

**Keywords:** antimicrobial, biofilm, *Listeria monocytogenes*, ozone, eco-friendly technology, foodborne pathogens, food processing environment, planktonic cells, food industries, food safety

## Abstract

Among food-borne pathogens, *Listeria monocytogenes* continues to pose concerns to food business operators due to its capacity to form biofilm in processing environments. Ozone may be an eco-friendly technology to control microbial contaminations, but data concerning its effect on *Listeria monocytogenes* biofilm are still limited. In this study, the effect of gaseous ozone at 50 ppm on planktonic cells and biofilm of reference and food-related *Listeria monocytogenes* strains was evaluated. Ozone caused a reduction in microbial loads of 3.7 ± 0.4 and 3.9 ± 0.4 Log10 CFU/mL after 10 and 30 min, respectively. A complete inactivation of planktonic cells after 6 h of treatment was observed. Biofilm inhibition and eradication treatments (50 ppm, 6 h) resulted in a significant decrease of the biofilm biomass for 59% of the strains tested, whilst a slight dampening of live cell loads in the biofilm state was observed. In conclusion, gaseous ozone is not sufficient to completely counteract *Listeria monocytogenes* biofilm, but it may be useful as an additional tool to contrast *Listeria monocytogenes* free-living cells and to improve the existing sanitization procedures in food processing environments.

## 1. Introduction

Despite the regular application of sanitization plans by food business operators (FBO), bacterial biofilms are commonly found in food processing environments [1]. Biofilms allow microorganisms to better resist harsh environmental conditions, causing negative effects in food facilities, including lower industrial operational efficiency and contaminations of the final product with consequent issues related to its shelf life and safety [2,3].

Among biofilm-forming food-borne pathogens, *Listeria monocytogenes* represents an important safety concern. *L. monocytogenes* is a ubiquitous Gram-positive bacterium responsible for human listeriosis [4], one of the most serious food-borne diseases with the highest case fatality (17.6%) and which showed a significant increasing trend in the last years [5]. This pathogen can survive and grow in a wide range of foods, such as dairy, meat, seafood and vegetable products, with a high incidence especially in ready-to-eat (RTE) foods [6,7]. The processing environment is considered the most likely source of foodstuffs contamination by *L. monocytogenes* as the pathogen, when organized in biofilm, can persist for months or even years on surfaces representing a source of recurrent contaminations [8,9,10,11,12,13].

In food processing industries, chemical biocides are commonly used in order to control microbial contamination and biofilm. However, there are several concerns related to the intensive and prolonged use of these substances. Long-term exposure to antimicrobial agents, for example, may increase the tolerance of microorganisms to certain compounds and lead to a phenomenon known as antimicrobial cross-resistance [14,15,16,17]. In addition, the most commonly used biocide products may have an environmental and human health impact [18]. Among the innovative anti-biofilm strategies [2,19,20,21,22], ozone is considered a promising eco-friendly technology as it spontaneously breaks down into oxygen and does not leave harmful residues on food contact surfaces or in the finished products [23,24,25]. With regard to this, ozone has a reduced environmental impact compared to other chemicals employed in food processing environments and its effectiveness against a wide range of microorganisms is well documented [18,26]. The future possible application of this technology in food processing environments has been thoroughly reviewed in the last years [26,27,28,29]. However, data concerning the action of ozone on microbial biofilm are still scarce. Few studies have investigated the effect of ozone treatment on sessile forms of *L. monocytogenes* [18,30,31,32]. Ozone molecules in gaseous state have a longer half-life and higher diffusion than the molecules in aqueous form [33]. This means that ozone gas may be used to control *L. monocytogenes* biofilm in hard-to-reach areas within food processing environments, such as niches and other “dead zones”, where the pathogen can persist [34]. Recently, several studies have highlighted that ozone gas may be effective in controlling microbial contamination and biofilms in the food industry, especially when used in high concentrations and for long treatment times [18,23,30,31]. In order to minimize health risks for operators due to the toxicity of ozone, this technology might be applied at the end of the production day, during the weekly closing days and in the absence of personnel [18,23].

Previous studies have shown that growth dynamics, biofilm formation abilities and stress resistance may vary among different strains belonging to *L. monocytogenes* species [35,36,37,38,39,40] due to the presence of several genes and/or accessory genetic elements, including plasmids, prophages, stress-survival islets, etc. [41,42,43,44,45]. In view of this strain variability, experiments by using reference and wild-type *L. monocytogenes* strains are needed to assess the potential effect of an anti-biofilm technology.

Therefore, the aim of this study was to evaluate the effectiveness of gaseous ozone (50 ppm) treatment against biofilm and planktonic cells among reference and food-related *L. monocytogenes* strains.

## 2. Materials and Methods

### 2.1. Bacterial Strains

A total of 22 *L. monocytogenes* isolates were included in this study (Table 1). Two reference strains (ATCC 19112, known for its adherence characteristics; ATCC 7644, known as a strong biofilm producer) [46,47] and 20 completely sequenced (whole genome sequencing) wild strains isolated from dairy environments (5), meat environments (5), dairy products (5) and meat products (5) in food industries located in Piedmont (Italy) (Collection of the Department of Veterinary Sciences, University of Turin) were used for experimental trials. 

### 2.2. Screening of Biofilm Forming Strains: Micro-Method Assays

The biofilm capacity of all strains was assessed by using the micro-method assay described by Stepanovic at al. [48]. Briefly, overnight cultures (37 °C) of each strain cultivated in brain heart infusion (BHI) broth (Oxoid, Milan, Italy) were diluted to obtain an optical density (OD) at 550 nm (Pharmacia Biotech Ultrospec-3000, Biochrom Ltd., Cambridge, UK) comparable to the 0.5 McFarland standard (cell concentration of about 8 Log10 CFU/mL). Subsequently, dilutions (1:100) of cultures were added in each well (0.2 mL in triplicate for each strain) of 96-well polystyrene microplates (Sarstedt, Nümbrecht, Germany), while the negative control wells contained the uninoculated broth. Microplates were then incubated at 37 °C for 24 h. After the incubation, the BHI broth was discarded and wells were washed thrice with 0.3 mL of sterile phosphate buffer saline solution (PBS, pH 7.3 ± 0.2; Oxoid). Biofilms were heat-fixed at 60 °C for 1 h and stained with 0.15 mL of a 2% *w*/*v* crystal violet solution (Chem-lab, Zedelgem, Belgium) for 15 min. After staining, the solution was removed, wells were washed with distilled water and dried at 37 °C for 15 min. To quantify the biofilm formation, 0.15 mL of 95% ethanol solution (Honeywell, Charlotte, NC, USA) were added to each well and the absorbance of the destaining solution was measured at 595 nm (iMark plate reader, Bio-Rad, Sydney, NSW, Australia). An average OD value was calculated for each strain (OD-S), while the optical density of the negative control (OD-C) was calculated by using the mean values of all negative control wells plus three standard deviations. The strains were classified as weak (OD-C < OD-S ≤ 2 × OD-C), moderate (2 × OD-C < OD-S ≤ 4 × OD-C), strong (4 × OD-C < OD-S) and no (OD-S ≤ OD-C) biofilm producers.

### 2.3. Biofilm Production Quantification: Macro-Method Assays 

#### 2.3.1. Biofilm Production Indices (BPIs)

The biofilm production index (BPI) of each strain was calculated by using the macro-method assay following Di Bonaventura et al. [9] with some modifications. In detail, overnight cultures (37 °C) of each strain in BHI broth (Oxoid) were washed thrice with a PBS solution (Oxoid), centrifugated (4000 rpm for 10 min; ALC Multispeed PK121, ALC International srl, Cologno Monzese, Italy) three times and then re-suspended in BHI broth (Oxoid). Cultures were diluted to reach an OD of approximately 0.125 at 550 nm (Pharmacia Biotech Ultrospec-3000, Biochrom Ltd.), corresponding to a cell concentration of about 8 Log10 CFU/mL. Three milliliters of each diluted culture were added (3 wells for each strain) to polystyrene tissue culture plates (growth area = 8.87 cm^2^; Sarstedt), then incubated at 37 °C for 24 h. After incubation, BHI broth (Oxoid) was removed using a sterile Pasteur pipette and each well was washed three times with 3 mL of sterile PBS (Oxoid) to eliminate non-adherent cells. The formed biofilm was fixed at 60 °C for 1 h and stained with 3 mL of a 2% crystal violet solution (95% ethanol, Honeywell; 2% crystal violet, Chem-lab) for 20 min. After staining, wells were washed three times with distilled water and dried at 37 °C for 15 min. Then, 3 ml of a 33% acetic acid (Merck, Darmstadt, Germany) solution were added to each well. After 20 min, 0.2 mL from each sample were transferred to a 96-well microtiter plate (Sarstedt) and the OD level of the destaining solution was measured at 490 nm. Results were normalized calculating the BPIs considering the growth area of each well (8.87 cm^2^) (Equation (1)):

(1) $$\mathrm{BPIs}=\left(\frac{\mathrm{ODmean}}{\mathrm{biofilm}\;\mathrm{surface}\;\left({\mathrm{mm}}^{2}\right)}\right)\times 1000$$

#### 2.3.2. Quantification of Viable Bacteria in the Biofilm

Viable bacteria in biofilm state (macro-method assay) were counted after mechanical scraping of adherent cells in each well. In detail, overnight cultures (37 °C) of each strain in BHI broth (Oxoid) were washed, centrifugated, diluted and added (3 wells for each strain) to polystyrene tissue culture plates (Sarstedt) as previously described (see Section 2.3.1). After incubation (37 °C for 24 h), BHI broth (Oxoid) was removed using a sterile Pasteur pipette and each well was washed thrice with 3 mL of sterile PBS (Oxoid) to eliminate non-adherent cells. Then, 3 ml of PBS (Oxoid) were added to each well and adherent cells were removed by scraping (mechanical action) as described by Zand et al. [49]. Bacterial suspensions were diluted (1:10) in sterile physiological saline peptone (PS) (0.85% NaCl, Carlo Erba, Italy; 0.1% Bacteriological Peptone, Oxoid) and inoculated by surface plating on BHI agar (Oxoid) plates, then incubated at 37 °C for 48 h. The *L. monocytogenes* counts were expressed as Log10 CFU/mL.

### 2.4. Ozonization Assays

#### 2.4.1. Treatment Chamber and Experimental Conditions

**Figure 1:**
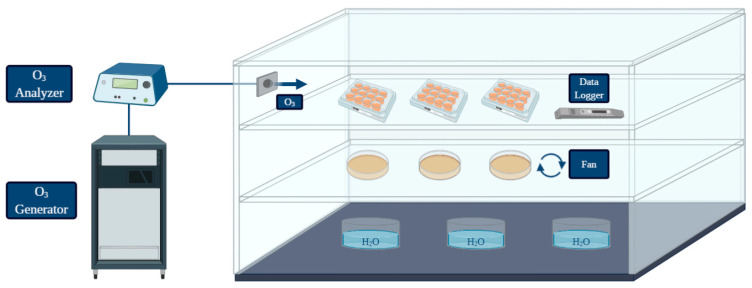
Graphical representation of the system used for the treatments performed in the present study (created with BioRender; https://biorender.com/; accessed on 27 May 2021).

An in vitro simulation system was designed to test the pathogen inactivation and the anti-biofilm activity of ozone (O_3_) gas under different treatment conditions. A graphical representation was created with BioRender (https://biorender.com/; accessed on 27 May 2021) [50] (Figure 1). The system was equipped with: O_3_ generator, O_3_ monitor and the source of oxygen and air gases used for O_3_ production. The ozone-inert plexiglass chamber (Biofresh Group Ltd., Northumberland, UK) was connected to an ozone generator (Model-LF5; Biofresh Group Ltd.) and gas injection was regulated by an ozone analyzer (UV-100, EcoSensor, Santa Fe, USA). A fan was placed in the chamber to allow a homogeneous distribution of the gas during each treatment. Containers with warm water were placed on the bottom in order to keep high (≥90%) relative humidity (RH), since the bactericidal effect of gaseous ozone seems to be linked with increasing RH (optimum level of 90–95%, no effect below 50%) [25]. All the experiments were performed at room temperature. A data logger (Testo 174 H, Testo AG, Lenzkirchen, Germany) was placed in the chamber to check temperature fluctuations and RH (%) during treatments. The ozone treatments were performed with a high gas concentration (50 ppm). The effect on *L. monocytogenes* planktonic cells was firstly assessed during two short treatment times (10 min and 30 min) since free cells are generally more susceptiblethan bacteria in biofilm, so short exposure times to high ozone concentrations should have caused a significant logarithmic reduction. Secondly, a long treatment (6 h) was carried out to test whether prolonged exposure could lead to a total inactivation of high planktonic cell loads. For the biofilm form, considering the literature data and the higher resistance to oxidative stress of cells in the sessile state compared to the planktonic forms [18,23,30,31,32], the ability of ozone gas to prevent and eradicate *L. monocytogenes* biofilm was tested only during a prolonged treatment time (6 h).

#### 2.4.2. Effect of Gaseous Ozone on *L. monocytogenes* Planktonic Cells

*L. monocytogenes* strains were pre-cultured in tryptic soy broth (TSB—Oxoid) at 37 °C overnight. Cultures were diluted (1:10) in sterile PS and appropriate dilutions were plated in duplicate on tryptic soy agar (TSA—Oxoid). Inoculated plates were submitted to treatments with gaseous ozone at 50 ppm for 10, 30 min and 6 h. Inoculated TSA plates not submitted to ozonization were control tests. To enumerate *L. monocytogenes* cells, treated and control plates were incubated at 37 °C for 24/48 h. Bacterial counts, before and after the treatments, were performed to evaluate the logarithmic reduction (Log10 CFU/mL) caused by ozone exposure.

#### 2.4.3. Biofilm Inhibition by Gaseous Ozone

This experimental step was aimed at evaluating the capacity of gaseous ozone to affect the biofilm formation abilities of the strains. For this purpose, bacteria were preliminary exposed to the ozone treatments before the incubation of plates and the subsequent biofilm formation. This possible preventive activity of ozone was assessed following the methods previously described (macro-method assay; see Section 2.3.1 and Section 2.3.2). In this case, the protocol was modified including an additional step (ozone gas treatment). Specifically, revitalized cultures were washed, centrifugated, resuspended, diluted and poured in polystyrene tissue culture plates (6-wells; Sarstedt). Plates were subjected to treatment with 50 ppm of gaseous ozone for 6 h and incubated at 37 °C for 24 h. After incubation, BPIs and counts of viable adherent cells in the biofilm state were carried out as described before. Finally, BPIs and loads of viable adherent cells calculated were compared to those detected after the biofilm production assay (controls) to evaluate the effect of exposure to ozone gas.

#### 2.4.4. Biofilm Eradication by Gaseous Ozone

These treatments were carried out to evaluate the effect of ozone gas on consolidated biofilms. Pre-formed biofilms of each strain were exposed to gaseous ozone to assess the eradication capacity of this technology. In this case, we also modified the previously described protocols (macro-method assay; see Section 2.3.1 and Section 2.3.2) including an additional step (ozone gas treatment). Revitalized cultures were washed, centrifugated, resuspended, diluted, poured in polystyrene tissue culture plates and incubated at 37 °C for 24 h to allow the biofilm formation. After incubation, 3 mL of BHI broth (Oxoid) were removed from each well and the cells organized in biofilm were exposed to 50 ppm of gaseous ozone for 6 h. After treatment, BPIs and viable adherent bacteria in the biofilm were quantified as described before. Finally, BPIs and loads of viable adherent cells calculated were compared to those detected after the biofilm production assay (controls) to evaluate the effect of exposure to ozone gas.

### 2.5. Statistical Analyses and Graphing

To evaluate the effect of O_3_ on the pathogen in planktonic form (see Section 2.4.2), data were analyzed performing a two-way analysis of variance (ANOVA) followed by a Tukey’s multiple comparison test (*p* < 0.05). As far as the anti-biofilm effect of O_3_ gas is concerned (see Section 2.4.3 and Section 2.4.4), significant differences in BPIs before and after the ozone treatments were calculated performing a two-way ANOVA followed by a Dunnett’s multiple comparison test (*p* < 0.05), while the Tukey’s multiple comparison test (*p* < 0.05) was applied to analyze the data on viable adherent cells before and after the ozone exposure. Statistical analyses and graphing were conducted with GraphPad Prism version 9.0.0 (GraphPad Software, San Diego, CA, USA).

## 3. Results

### 3.1. Screening of Biofilm Forming Strains: Micro-Method Assays

All *L. monocytogenes* were previously classified as biofilm-forming strains. In this study, 18% (4/22) were strong biofilm producers, 18% (4/22) moderate biofilm producers and 64% (14/22) weak biofilm producers.

### 3.2. Effects of O_3_ on L. monocytogenes Planktonic Cells

A reduction of *L. monocytogenes* loads was observed after the short-term (10 and 30 min) and long-term (6 h) treatments with gaseous ozone at 50 ppm compared to control samples (Table 2). Specifically, 10 min of treatment caused a mean logarithmic reduction of 3.7 ± 0.4 Log10 CFU/mL, while the mean logarithmic reduction of *L. monocytogenes* loads after 30 min of treatment was 3.9 ± 0.4 Log10 CFU/mL. Significant differences in loads reduction between the two short-term treatments (10 and 30 min) were observed for strain n.18 and n.20. The long-term exposure (6 h) led to a total inactivation of 17 strains (77.3% of the 22 strains tested). Only for five strains (n.17, n.38, n.40, ATCC 7644, ATCC 19112; 22.7% of all strains) colonies grew in plates treated for 6 h, while for the remaining strains loads were below the detection limit (1 Log10 CFU/mL).

### 3.3. Effect of O_3_ on Biofilm

#### 3.3.1. Effect on the Biofilm Biomass

**Figure 2:**
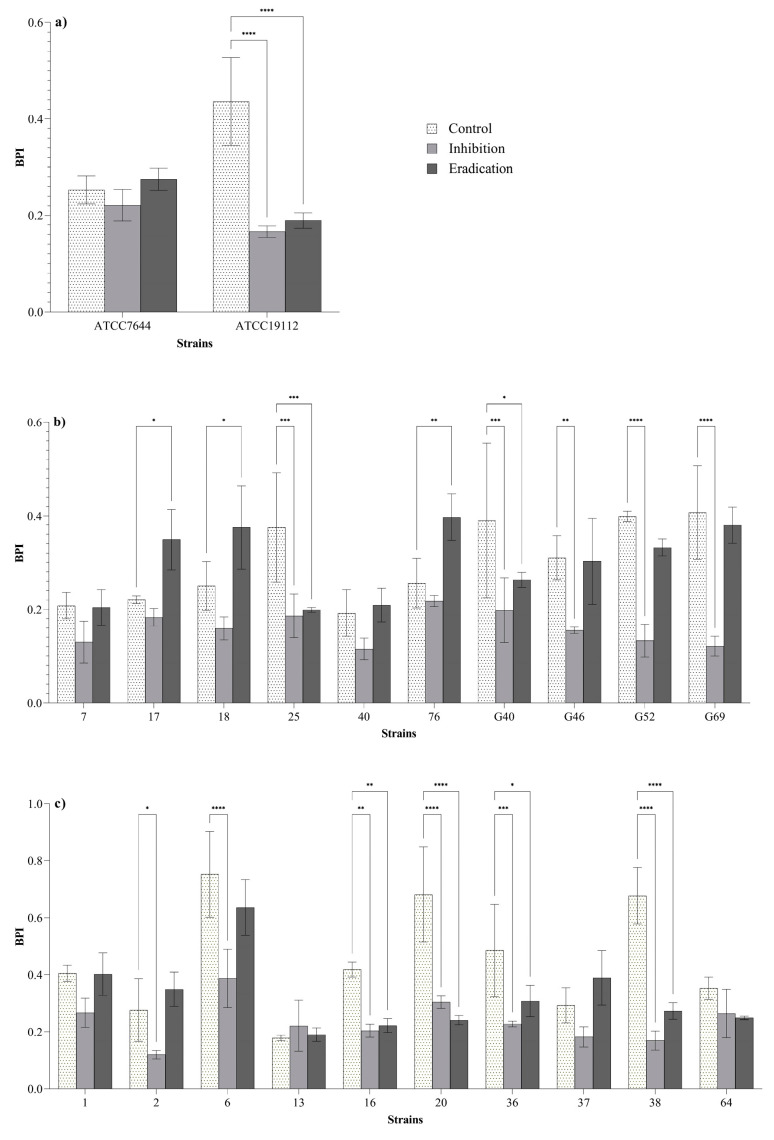
Biofilm production indices (BPIs) of ATCC (**a**), dairy-related (**b**) and meat-related (**c**) *L. monocytogenes* strains after the inhibition and eradication treatments (50 ppm of gaseous ozone for 6 h) compared to control samples. Error bars indicate the standard deviation between three replicates. Asterisks indicate the significant differences according to the Dunnett’s multiple comparison (*p* < 0.05).

BPIs of 13 strains (59% of the 22 strains tested) were significantly lower after the inhibition and/or eradication treatments compared to the control BPIs (Figure 2). Regarding the inhibition treatments (bacteria exposed to ozone before the biofilm formation; see Section 2.4.3), a reduction of BPIs was observed in 27% (6/22) of *L. monocytogenes* strains, suggesting a reduced biofilm formation ability of these isolates after the preliminary exposure to ozone gas. An effect in both of inhibition and eradication (pre-formed biofilms exposed to ozone gas; see Section 2.4.4) of biofilm was observed in 32% (7/22) of *L. monocytogenes* strains. No effect was observed in 41% (9/22) of *L. monocytogenes* strains. Three strains showed significantly higher BPIs after the eradication treatments compared to the BPIs of the control.

#### 3.3.2. Effect on Viable Biofilm-Detached Cells

Outcomes of viable adherent cells counts after the ozone exposure compared to control samples are given in Table 3. After the inhibition treatments (bacteria exposed to ozone before the biofilm formation; see Section 2.4.3), the mean reduction of viable adherent cells was 0.7 ± 0.4 Log10 CFU/mL. After the eradication treatments (pre-formed biofilms exposed to ozone gas; see Section 2.4.4), instead, a mean reduction of 0.8 ± 0.5 Log10 CFU/mL was detected. Significant differences in logarithmic reductions among inhibition and eradication treatments were observed for 37% (8/22) of *L. monocytogenes* strains.

## 4. Discussion

Previous studies showed that the antimicrobial efficacy of treatments with gaseous ozone against planktonic and sessile cells is affected by the concentration and the treatment time. In regard to this, Guzzon et al. [51] demonstrated that prolonged exposure (up to 6 h) to gaseous ozone reduced the total microbial loads on wooden shelves used for the ripening of traditional Italian cheeses. A recent study carried out by Botta et al. [23] in slaughterhouse environments showed that ozone gas could be an efficient adjunct sanitizing method if used at concentrations of 20 and 40 ppm for 12 h. As for *L. monocytogenes*, previous research reported that high levels of ozone are required to obtain an effect against biofilms formed by this pathogen. Nicholas et al. [31] observed a mean reduction of 3.41 Log10 CFU/cm^2^ for *L. monocytogenes* cells attached to stainless steel after a 1 h treatment with 45 ppm of gaseous ozone, while a reduction less than 1 Log10 CFU/cm^2^ was detected with 10 ppm of ozone at the same time. On the contrary, the same microorganisms organized in biofilm were significantly more resistant after a treatment with ozone gas at 45 ppm for 1 h. Recently, Harada et al. [30] demonstrated the efficiency of gaseous ozone at high concentration (45 ppm) as a dry sanitizing method against *L. monocytogenes* biofilm.

Based on these literature data, we decided to perform our experiment with a high ozone gas concentration (50 ppm) in order to evaluate its effect against *L. monocytogenes* isolates from food industries. Firstly, the effect of ozone gas for 10 and 30 min was assessed against *L. monocytogenes* planktonic cells. Both short exposure times at 50 ppm resulted in a significant logarithmic reduction for all strains tested. After this step, the strains were exposed to ozone gas (50 ppm) for 6 h. This long treatment time resulted in a total inactivation of planktonic cells (see Section 3.2 and Table 2). These findings suggest that high concentrations of ozone in gaseous form may be applied to destroy *L. monocytogenes* planktonic cells in hard-to-reach areas within food processing environments. Therefore, a significant antimicrobial effect (reduction of 3.7 ± 0.4 Log10 CFU/mL and 3.9 ± 0.4 Log10 CFU/mL) can be achieved even with short treatment times (10 and 30 min).

Considering that bacteria in biofilm state are known to express a higher resistance to oxidative stress compared to the planktonic forms [18,23,30,31,32], biofilm inhibition and eradication tests were performed at 50 ppm for 6 h. All strains were previously classified as biofilm producers by using 96-well microtiter plates (micro-method assay; see Section 2.2 and Section 3.1). In order to assess the effect of ozone gas treatment in prevention (biofilm-inhibition) and removal (biofilm-eradication) of biofilm, we used different methods to quantify the biofilm biomass and the loads of viable biofilm-detached cells (see Section 2.4.3 and Section 2.4.4). In these experiments, the 6-well plates were selected to overcome the limitation of the basic microtiter plate (96-wells format) concerning the possible nutrient limitation and to maximize the exposure of sessile cells to ozone gas. In addition, we attempted to investigate the inhibition and eradication effect of ozone gas in conditions simulating those of food processing plants. With regard to this, the tests were carried out in BHI broth during the inhibition assay (see Section 2.4.3) and without drying the pre-formed biofilm in the eradication assay (see Section 2.4.4) while keeping the residual BHI broth in the wells. Organic matter, in fact, may persist on surfaces or niches after routine cleaning and disinfection activities in food processing environments. The response of *L. monocytogenes* in biofilm state compared to planktonic form (see Section 3.3 and Figure 2) was different. As previously reported by other authors, the bacteria organized in biofilm can increase their resistance to oxidative stress [18,32]. Regarding reference strains (strong biofilm producers), ozone exposure resulted in significant reductions of BPIs after the inhibition and eradication assays for strain ATCC 19112, whereas no effect was observed for strain ATCC 7644 (Figure 2a). Ozone gas caused significant reductions in BPIs after inhibition and/or eradication assays in 60% (6/10) of isolates from meat products and meat processing environments (Figure 2c). As for *L. monocytogenes* isolates from dairy products and dairy processing environments, significant reductions in BPIs after inhibition and/or eradication assays were observed for 50% (5/10) of the strains (Figure 2b). The results seem to suggest that dairy-related strains may be more resistant to oxidative stress than meat-related isolates. Environmental strains showed a higher tolerance to ozone exposure than food isolates (Figure 2b,c). The latter outcome appears justifiable, since it has been demonstrated that environmental strains of *L. monocytogenes* can persist in food processing plants for months or years, developing a greater resistance to different types of stress and several antimicrobial compounds [10,15,16,17,36].

In order to evaluate the potential correlation between the reduction in BPIs and viable biofilm-detached cells after the ozone treatment, counts of adherent bacteria in established biofilms were performed. Outcomes showed a slight reduction of microbial loads for all strains after the ozone treatment compared to control samples. The cell reduction was not correlated with decreases in BPIs (see Section 3.3.2 and Table 3). As previously reported by several authors, the biofilm biomass is not linked to bacterial viability. The total biomass, as measured using the crystal violet staining method, includes live, un-culturable, dead bacteria and the extracellular polymeric matrix [52,53,54]. The lower BPIs observed for some strains after the inhibition assay may indicate a reduced capacity of cells to produce the extracellular polymeric matrix after a preliminary exposure to oxidative stress. Similarly, BPIs obtained after the eradication of pre-formed biofilm indicated that the oxidative stress caused a significant reduction of the total biomass in biofilm with probable structural losses in the extracellular matrix. For three strains (n.17, n.18, n.76), BPIs were significantly higher than BPIs of the control after the eradication assays. We hypothesized that these strains developed a resistance as a response to the oxidative stress in the first hours during the exposure to ozone, increasing the production of extracellular matrix. As reported by Abeysundara et al. [55], some *L. monocytogenes* strains can increase their survival capacity at lethal oxidative stress when cells are preliminary exposed to sub-lethal oxidative stress. This phenomenon is certainly worrying and should be emphasized, even though it occurred only for three strains (13.6% of the strains tested). Studies on more isolates are needed to understand the mechanisms behind this adaptation. This aspect is crucial to avoid that strains highly resistant to oxidative stress may be selected as a consequence of ozone treatments in the processing environments.

Our findings led to some discussion points on the real effectiveness of gaseous ozone against *L. monocytogenes* biofilm. Ozone gas was effective in reducing *L. monocytogenes* planktonic cells, whereas its action on these bacteria organized in biofilm seems limited and strain specific. To date, the great challenge in the food industry is to prevent and/or remove established *L. monocytogenes* biofilm. Our outcomes emphasized the limits of the use of ozone gas to mitigate *L. monocytogenes* biofilm. Regarding the methods used in our experiment, an indication on the effect of ozone gas against the total biofilm biomass can be obtained by using colorimetric staining assays. The reductions in BPIs were not linked to loads of viable biofilm-detached cells. Reductions in BPIs should certainly be taken in high regard since they indicate a potential action of ozone gas on the extracellular matrix which protects live cells from several stressful conditions (such as oxidative stress) [54]. However, counts of viable biofilm-detached cells demonstrated that bacteria could resist after substantial damages on the polymeric matrix keeping similar loads after ozone treatments. These results indicate that the application of gaseous ozone is not sufficient to counteract *L. monocytogenes* biofilm. Probably, ozone gas could be more effective on mixed-species biofilms. In the food environment, *L. monocytogenes* can persist in dual-species biofilm with other bacterial genera, such as *Pseudomonas* spp. [56]. Considering the high activity of gaseous ozone against *Pseudomonas* spp. [57] and other food-related bacteria, its application may act selectively against one population in mixed-species biofilms causing damage on the biofilm structure and a subsequent loss of protection for the bacterial population less sensitive to the oxidative stress. In addition, the results obtained on planktonic forms suggest that a preventive application of ozone gas may be useful to avoid the organization in biofilm by *L. monocytogenes* free-living cells. In light of these findings and according to other authors [18,23], gaseous ozone could be useful as an additional tool to improve the existing cleaning and disinfection procedures to control microbial contamination in food processing environments.

In general, despite the unquestionable advantages of gaseous ozone as an antimicrobial agent with a low environmental impact, there are several limits regarding the practical application of this technology as an anti-biofilm agent in food processing plants. These concerns arise from the high concentrations and the long exposure time required to achieve a significant effect on *L. monocytogenes* biofilm [18,30,31]. High ozone levels, indeed, are dangerous for human health, so its potential application in food industries would be possible only during downtime periods and in the absence of operators [18]. In regard to this, the application of ozone gas for the disinfection of empty cheese ripening rooms has been approved by the Italian Ministry of Health [57]. Recently, Botta et al. [23] carried out ozone gas treatments in high concentrations (4, 20 and 40 ppm) during the weekly closing days in red meat processing environments [23]. Another limitation of ozone treatment could be linked to the relative humidity of processing environments and foodstuffs. The bactericidal effect of gaseous ozone seems to be linked with high relative humidity [25], so this technology may be more effective in high humidity environments, while its action may be milder in low humidity conditions. Finally, although plastic materials commonly used in food industries, such as PTFE (Teflon), PVDF (Kynar), PVC (rigid and flexible) and ECTFE (Halar) exhibited good resistance to corrosion during the ozone exposure [27], further studies are needed to assess the potential effect of this technology on equipment after long treatment times.

## 5. Conclusions

Ozone gas in high concentration (50 ppm) was effective on *L. monocytogenes* planktonic cells, whereas its action in prevention and removal of *L. monocytogenes* biofilm was partial, strain-dependent and limited to the total biofilm biomass with a minimal effect on live adherent cells in the biofilm state. High concentrations of ozone gas are not sufficient to counteract *L. monocytogenes* biofilm. However, our results suggest that ozone gas may be applied as an additional tool against *L. monocytogenes* planktonic cells and to improve the existing sanitization procedures in food processing environments.

## Figures and Tables

**Table 1:** Characteristics of *L. monocytogenes* strains used in the present study.

Category	Strain ID (Internal)	Strain ID (NCBI) ^1^	Source	Lineage	Serogroup	Sequence Type (ST)	Clonal Complex (CC)
ATCC	ATCC 7644	ATCC 7644	Human	II	IIc	122	9
	ATCC 19112	WSLC1001	Human	II	IIc	12	7
Dairy	17	CFSAN045778	Product	II	IIa	9	9
	25	CFSAN045791	Product	II	IIc	9	9
	76	CFSAN044775	Product	II	IIc	9	9
	G40	CFSAN044840	Product	II	IIa	325	31
	G52	CFSAN044807	Product	II	IIa	325	31
	7	CFSAN045850	Production Environment	II	IIa	325	31
	18	CFSAN045794	Production Environment	II	IIa	9	9
	40	CFSAN044857	Production Environment	II	IIa	325	31
	G46	CFSAN044805	Production Environment	II	IIa	325	31
	G69	CFSAN044813	Production Environment	II	IIa	325	31
Meat	16	CFSAN045938	Product	II	IIa	325	31
	20	CFSAN045829	Product	II	IIa	9	9
	36	CFSAN044741	Product	II	IIc	9	9
	38	CFSAN044748	Product	II	IIc	9	9
	64	CFSAN044767	Product	II	IIc	9	9
	1	CFSAN046012	Production Environment	II	IIc	9	9
	2	CFSAN045995	Production Environment	II	IIa	325	31
	6	CFSAN045858	Production Environment	II	IIc	9	9
	13	CFSAN045971	Production Environment	II	IIa	9	9
	37	CFSAN046048	Production Environment	II	IIc	9	9

^1^ ID of the strains in the NCBI database (https://www.ncbi.nlm.nih.gov/; accessed on 20 May 2021).

**Table 2:** Logarithmic reduction (Log10 CFU/mL) of *L. monocytogenes* planktonic cells after the ozone exposure (O_3_ = 50 ppm; 10, 30 min and 6 h) compared to control samples.

Strain ID	Logarithmic Reduction ^1,2^		
	10 min	30 min	6 h
ATCC 7644	3.2 ± 0.1 ^a^	3.6 ± 0.3 ^a^	7.2 ± 0.1 ^b^
ATCC 19112	3.1 ± 0.2 ^a^	3.1 ± 0.1 ^a^	7.5 ± 0.3 ^b^
1	3.1 ± 0.3 ^a^	3.5 ± 0.3 ^a^	≥7.7 ± 0.1 ^b^
2	3.9 ± 0.1 ^a^	4.3 ± 0.1 ^a^	≥9.2 ± 0.0 ^b^
6	4.2 ± 0.1 ^a^	4.4 ± 0.1 ^a^	≥9.2 ± 0.1 ^b^
7	3.7 ± 0.2 ^a^	3.9 ± 0.1 ^a^	≥9.0 ± 0.1 ^b^
13	4.1 ± 0.1 ^a^	4.0 ± 0.2 ^a^	≥9.2 ± 0.1 ^b^
16	3.3 ± 0.2 ^a^	3.3 ± 0.1 ^a^	≥8.2 ± 0.0 ^b^
17	3.6 ± 0.0 ^a^	3.5 ± 0.1 ^a^	7.4 ± 0.1 ^b^
18	4.5 ± 0.1 ^b^	4.0 ± 0.2 ^a^	≥9.1 ± 0.1 ^c^
20	3.6 ± 0.1 ^a^	4.2 ± 0.1 ^b^	≥8.4 ± 0.0 ^c^
25	3.5 ± 0.4 ^a^	3.5 ± 0.1 ^a^	≥8.2 ± 0.1 ^b^
36	3.3 ± 0.1 ^a^	3.7 ± 0.1 ^a^	≥8.7 ± 0.1 ^b^
37	4.1 ± 0.1 ^a^	4.2 ± 0.0 ^a^	≥9.2 ± 0.0 ^b^
38	4.1 ± 0.3 ^a^	4.1 ± 0.3 ^a^	7.2 ± 0.4 ^b^
40	4.1 ± 0.3 ^a^	3.9 ± 0.1 ^a^	8.2 ± 0.1 ^b^
64	3.7 ± 0.2 ^a^	4.1 ± 0.4 ^a^	≥8.8 ± 0.2 ^b^
76	3.7 ± 0.2 ^a^	4.1 ± 0.1 ^a^	≥8.9 ± 0.1 ^b^
G40	3.5 ± 0.2 ^a^	3.5 ± 0.1 ^a^	≥8.6 ± 0.1 ^b^
G46	4.1 ± 0.2 ^a^	4.2 ± 0.1 ^a^	≥9.1 ± 0.1 ^b^
G52	4.1 ± 0.2 ^a^	4.3 ± 0.2 ^a^	≥9.1 ± 0.2 ^b^
G69	3.9 ± 0.0 ^a^	4.1 ± 0.1 ^a^	≥9.1 ± 0.0 ^b^

^1^ Average ± Standard deviation of two replicates. ^2^ Values followed by different small letters in the same row are significantly different according to Tukey’s multiple comparison test (*p* < 0.05).

**Table 3:** Logarithmic reduction (Log10 CFU/mL) of *L. monocytogenes* viable adherent cells after the inhibition and eradication of the biofilm forms (O_3_ = 50 ppm; 6 h) compared to control samples.

Strain ID	Logarithmic Reduction ^1,2^	
	Inhibition	Eradication
ATCC 7644	0.6 ± 0.1	0.6 ± 0.1
ATCC 19112	0.7 ± 0.1 ^b^	1.2 ± 0.1 ^a^
1	1.0 ± 0.0	1.1 ± 0.0
2	1.2 ± 0.1	1.1 ± 0.1
6	0.4 ± 0.1 ^a^	0.1 ± 0.1 ^b^
7	1.3 ± 0.1	1.3 ± 0.1
13	0.2 ± 0.1	0.2 ± 0.1
16	1.1 ± 0.1	1.2 ± 0.1
17	0.6 ± 0.1 ^b^	0.9 ± 0.1 ^a^
18	1.6 ± 0.0	1.7 ± 0.1
20	0.6 ± 0.0 ^a^	0.1 ± 0.0 ^b^
25	0.9 ± 0.0	1.0 ± 0.0
36	0.9 ± 0.1 ^b^	1.2 ± 0.1 ^a^
37	0.7 ± 0.1	0.6 ± 0.1
38	0.7 ± 0.1 ^a^	0.3 ± 0.2 ^b^
40	0.1 ± 0.1	0.2 ± 0.1
64	0.6 ± 0.1	0.4 ± 0.1
76	0.6 ± 0.1 ^b^	1.3 ± 0.2 ^a^
G40	0.9 ± 0.0	1.1 ± 0.2
G46	0.4 ± 0.1	0.6 ± 0.1
G52	1.0 ± 0.1	1.0 ± 0.1
G69	0.4 ± 0.2 ^a^	0.0 ± 0.1 ^b^

^1^ Average ± Standard deviation of three replicates. ^2^ Values followed by different small letters in the same row are significantly different according to the Tukey’s multiple comparison test (*p* < 0.05). Strain n. 20 = highest significant difference in logarithmic reduction in inhibition compared to eradication.
